# Application value of real-time 3D speckle tracking imaging in left atrial function evaluation of patients with paroxysmal atrial fibrillation

**DOI:** 10.1097/MD.0000000000038206

**Published:** 2024-05-24

**Authors:** Yufeng Wang, Dong Bai, Xiaojun Lu, Haijun Hou, Lei Liang

**Affiliations:** a Department of Ultrasound, Aerospace Center Hospital, Beijing, China; b Department of Radiology, Aerospace Center Hospital, Beijing, China.

**Keywords:** 3D speckle tracking technology, left atrial function, paroxysmal atrial fibrillation, strain rate

## Abstract

**Objective:**

To evaluate left atrial volume and function in patients with paroxysmal atrial fibrillation (AF) combined with left atrial appendage thrombosis and patients with paroxysmal AF without left atrial appendage thrombosis by 3-dimensional speckle tracking imaging (3D-STI), and to explore the application value of this set of parameters in the evaluation of left atrial function in patients with paroxysmal AF.

**Materials and Methods:**

A total of 40 patients with paroxysmal AF admitted from December 2018 to December 2020 were selected as the observation group. All patients with paroxysmal AF in the observation group underwent transesophageal echocardiography. According to the presence of left atrial appendage thrombosis, the patients were divided into the AF without thrombosis group (24 cases) and the AF with thrombosis group (16 cases). Thirty normal people were selected as control group who were chosen as having no heart-related disease. The left atrial volume parameters (Left atrial maximum volume LAVmax, Left atrial minimum volume LAVmin, Left atrial volume before atrial contraction LAVpre-A, Left atrial stroke volume LAEV), left atrial ejection fraction (LAEF) and left atrial strain parameters (Left atrial reservoir longitudinal strain LASr, Left atrial conduit longitudinal strain LAScd, Left atrial contraction longitudinal strain LASct, Left atrial reservoir circumferential strain LASr-c, Left atrial conduit circumferential strain LAScd-c, Left atrial contraction circumferential strain LASct-c) of the 3 groups were measured by 3D-STI.

**Results:**

With the progression of paroxysmal AF, the left atrial volume increased, and the reservoir, conduit and contractile function were damaged. The left atrial volume continued to increase, and the reservoir, conduit and contractile function further decreased significantly in patients with AF combined with left atrial appendage thrombosis. LAEF was positively correlated with LASr and LASr_c.

**Conclusion:**

Real-time 3-dimensional spot tracking imaging (3D-STI) can evaluate the changes in left atrial volume and function in patients with paroxysmal AF, and has a certain reference value for clinical judgment of disease progression and prognosis.

## 1. Introduction

Atrial fibrillation (AF) is a disease with abnormal electrical origin and conduction function of atrial ectopic, which can lead to serious complications such as thromboembolism and reduced cardiac function. Studies show that the number of patients with AF is increasing year by year, and the death rate is also on the rise.^[1]^ Stroke is the most serious complication caused by AF, which often leads to high mortality and disability. With the development of 3-dimensional echocardiography technology, 3-dimensional speckle tracking imaging technology (3D-STI) can break through the limitation of 2-dimensional plane and analyze the entire left atrial function by tracking speckles in 3-dimensional space, so as to evaluate the left atrial function more clearly.^[2]^The aim of this study is to evaluate the changes of left atrial volume and function in patients with AF by using 3D-STI, to find the characteristics and rules of abnormal changes of left atrial, and to preliminarily evaluate the left atrial function in patients with AF, and to provide the basis for clinical prevention and treatment as soon as possible.

## 2. Materials and methods

### 2.1. Research object

A total of 40 patients with paroxysmal AF who were hospitalized in our hospital from December 2018 to December 2020 were collected, and 40 patients with paroxysmal AF were finally included. Inclusion criteria: patients clinically diagnosed as paroxysmal AF; The patient had a complete medical history and clinical data. Tolerated and underwent transesophageal echocardiography. Exclusion criteria: Patients with rheumatic mitral stenosis, valve replacement and other conditions; heart failure assessed by clinical symptoms and left ventricular ejection fraction, angina pectoris or myocardial infarction; Patients in the stage of AF at the time of image collection; those with unevaluable ultrasonographic images which cannot be used to assess, inability to cooperate with the examination, and inability to obtain informed consent; Patients with malignant tumors.

Transesophageal echocardiography was performed in all patients with paroxysmal AF. According to the examination results, the patients were divided into AF group without thrombus (24 cases) and AF group with thrombus (16 cases). In addition, 30 volunteers with normal ECG, ultrasound and other routine examinations and matched age and gender were selected as the control group. All subjects in the 3 groups underwent 2D and 3D echocardiography and 4D Auto LAQ analysis. All patients were treated with warfarin regular anticoagulant therapy. The studies involving human participants were reviewed and approved by the Ethics Committee of our hospital. Written informed consent to participate in this study was provided by the patient.

### 2.2. Instruments and methods

**Figure 1.**
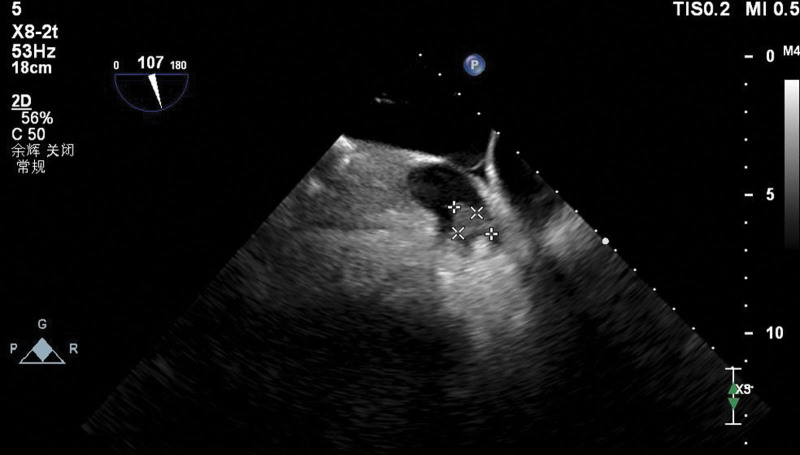
Left atrial appendage thrombus on transesophageal echocardiography in AF patients with thrombus group. AF = atrial fibrillation.

All the patients enrolled with paroxystal AF were examined with Philips EPIQ7 ultrasound, X8–2T intracavity ultrasound probe, and transesophageal echocardiography (TEE) was performed, and 0°, 45°, 90°, and 135° were determined in the middle section of the esophagus. The thrombus of left atrium or left auricle was observed (Fig. 1).

Blood pressure, height, weight and other routine clinical parameters were recorded in the 3 groups, respectively. Vivid E9 cardiac ultrasonic diagnostic machine produced by GE was used for 2-dimensional and 3-dimensional echocardiographic examination with M5S 2D ultrasonic probe with a frequency of 1.5 to 4.5MHz and 4V 3D ultrasonic probe with a frequency of 1.7 to 3.3MH. The frame rate was adjusted to the range of 25 to 40 frames/s, and EchoPAC 203 software was used for offline image analysis. Electrocardiogram was connected throughout the examination.

Two-dimensional echocardiographic data collection: The subject was asked to lie on the left side in a state of calm breathing, and the left atrial anterior and posterior diameter (LAd), right ventricular end-diastolic diameter (RVd), left ventricular end-diastolic diameter (LVd), and left ventricular ejection fraction (LVEF) were measured with M5S probe.

**Figure 2.**
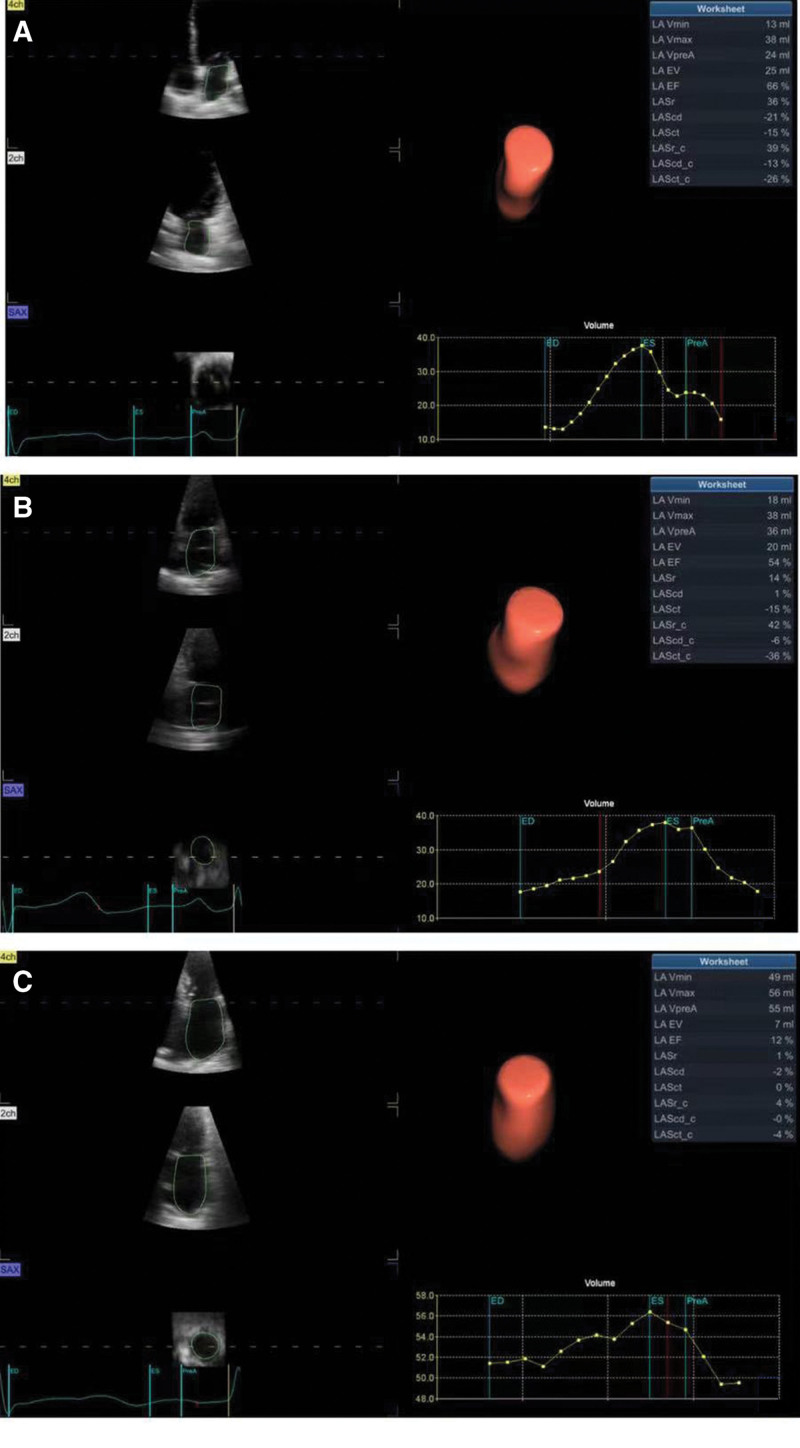
Three-dimensional images of the left atrium, associated volume and strain parameters between each group. (A) Three-dimensional images, relevant volume and strain parameters of left atrium in control group. (B) Three-dimensional images, relevant volume and strain parameters of left atrium in the non-thrombotic atrial fibrillation group. (C) Three-dimensional image, relevant volume and strain parameters of left atrium in patients with atrial fibrillation and thrombus.

Three-dimensional echocardiography images and data acquisition: The subject was instructed to hold his breath at the end of exhalation, the left ventricle was placed in the center of the image with a 4V probe, the apical 4-chamber section with clear intimal surface was selected, and real-time full-volume images of the heart were continuously collected for 3 cardiac cycles. The images were imported into EchoPAC203 software for offline analysis, the sampling points were placed in the middle of the mitral valve opening, and the sampling points were ensured to be placed in the middle of the mitral valve opening in 3 planes, and the 4D Auto LAQ program was entered to measure the relevant parameters of the left atrium. Volume parameters: left atrial minimum volume (LAVmin), left atrial maximum volume (LAVmax), left atrial volume before atrial contraction (LAVpre-A), left atrial stroke volume (LAEV), left atrial ejection fraction (LAEF); Strain parameters: left atrial reservoir longitudinal strain (LASr), left atrial conduit longitudinal strain (LAScd), left atrial contraction longitudinal strain (LASct), left atrial reservoir circumferential strain (LASr-c), left atrial conduit circumferential strain (LAScd-c), left atrial contraction circumferential strain (LASct-c) during systolic, early diastolic and late diastolic. Among them, LAEF, LASr, and LASr-c reflect the storage function of the left atrium, LAScd and LAScd-c reflect the duct function of the left atrium, and LASct and LASct-c reflect the active contraction function of the left atrium^[3]^ (Fig. 2).

In order to accurately collect the data of patients, the cardiac cycles with a relatively regular rhythm between AF episodes were selected, and each value was selected for 3 cardiac cycles to obtain the mean value.

### 2.3. Statistical methods

SPSS 23.0 statistical software was used for statistical analysis of the data. Those with normal distribution of measurement data were represented by mean ± standard deviation (x ± s), F-test was used for comparison among the 3 groups, and LSD-t test was used for further pair comparison. *P* < .05 was considered statistically significant. Counting data were expressed as percentage (%), Chi-square test was used to compare inter-group rates, and *P* < .05 was considered statistically significant. Univariate correlation analysis was performed by Pearson correlation analysis, and *P* < .05 was considered statistically significant. Intra-observer repeatability and inter-observer consistency were evaluated using intra-group correlation coefficients. *P* < .05 was considered statistically significant.

## 3. Results

### 3.1. Three groups of basic clinical data comparison between the research object

There were no significant differences in age, sex, BMI (kg/m^2^), hypertension or diabetes (*P* > .05) (Table 1).

**Table 1:** Comparison of basic clinical data of the 3 groups.

	Control group (n = 30)	Atrial fibrillation without thrombus group (n = 24)	Atrial fibrillation with thrombus group (n = 16)	*P* value
Age (yr)	62.1 ± 10.77	62.3 ± 12.69	62.7 ± 8.59	0.0828
Sex (men/women)	18/12	13/11	11/5	.0923
BMI* (kg/m^2^)	22.6 ± 7.0	23.5 ± 4.9	23.8 ± 5.8	.0654
Hypertension (Yes/No)	9/21	11/13	9/7	.0724
Diabetes (Yes/No)	6/24	8/16	6/10	.0825

* BMI stands for body mass index, BMI = weight/height squared (international units kg/m^2^).

### 3.2. Comparison of 2-dimensional echocardiographic parameters among the 3 groups

Compared with the control group, although LAd and LVd increased and LVEF decreased in AF group without thrombus, the differences were not statistically significant (all *P* > .05). Compared with control group, LAd and LVd increased and LVEF decreased in AF group with thrombus, the difference was statistically significant (*P* < .05 for all). Compared with AF without thrombus, LAd and LVd increased and LVEF decreased in the group with thrombus, and the differences were statistically significant (*P* < .05 for all) (Table 2). Intra-observer repeatability and inter-observer consistency are good, intra-group correlation coefficients as 0.999 and 0.997.

**Table 2:** Comparison of 2-dimensional echocardiographic parameters among the 3 groups.

	Control group (n = 30)	Atrial fibrillation without thrombus group (n = 24)	Atrial fibrillation with thrombus group (n = 16)	F value	*P* value
LAd (mm)	31.56 ± 2.97	35.15 ± 5.51	43.10 ± 6.21*^,^**	14.72	<.01
RVd (mm)	21.33 ± 2.01	21.40 ± 2.24	23.40 ± 5.72	2.39	.0829
LVd (mm)	43.40 ± 6.17	44.00 ± 7.69	49.75 ± 7.16*^,^**	5.637	<.01
LVEF (%)	66.30 ± 3.02	64.00 ± 3.63	56.85 ± 3.14*^,^**	17.57	<.01

Compared with control group,

* *P* < .05; Compared with atrial fibrillation group without thrombus,

** *P* < .05.

LAd = left atrial anterior and posterior diameter, LVd = left ventricular end-diastolic diameter, LVEF = left ventricular ejection fraction, RVd = right ventricular end-diastolic diameter.

### 3.3. Comparison of 3-dimensional echocardiographic left atrial volume index and functional parameters among the 3 groups

Compared with control group, LAVmin, LAVmax and LAVpre-A in AF group without thrombus increased, but the difference was not statistically significant (all *P* > .05). The absolute values of LAEF and LAScd, LASct, LAScd-c, LASct-c, LASr and LASr-c decreased, and the differences were statistically significant (all *P* < .05). Compared with the control group, LAVmin, LAVmax and LAVpr-eA in AF group with thrombus increased, while absolute values of LAEF and LAScd, LASct, LASct-c, LASct-c, LASr, and LASr-c decreased, with statistical significance (all *P* < .05). Compared with the group without AF thrombus, LAVmin, LAVmax, and LAVpre-A in the group with AF thrombus further increased, while the absolute values of LAEF, LAScd, LASct, LASct-c, LASr, and LASr-c further decreased, with statistical significance (all *P* < .05) (Table 3).

**Table 3:** Comparison of 3-dimensional echocardiographic left atrial volume index and functional parameters among the 3 groups of subjects.

	Control group (n = 30)	Atrial fibrillation without thrombus group (n = 24)	Atrial fibrillation with thrombus group (n = 16)	F value	*P* value
LAVmin (mL)	15.67 ± 4.97	23.70 ± 12.25	58.10 ± 15.26*^,^**	36.35	<.01
LAVmax (mL)	37.13 ± 9.72	46.05 ± 10.87	75.65 ± 16.99*^,^**	16.06	<.01
LAVpreA (mL)	26.10 ± 7.86	33.95 ± 11.37	67.40 ± 14.39*^,^**	23.78	<.01
LAEV (mL)	21.60 ± 5.76	22.55 ± 7.18	19.45 ± 8.15	0.87	.0635
LAEF (%)	57.80 ± 6.89	49.60 ± 8.02^a^	25.00 ± 5.97*^,^**	35.40	<.01
LASr (%)	25.10 ± 5.25	18.75 ± 7.26^a^	6.00 ± 3.92*^,^**	29.78	<.01
LAScd (%)	−(13.60 ± 4.64)	−(10.40 ± 4.68)^a^	−(6.30 ± 3.88)*^,^**	12.64	<.01
LASct (%)	−(12.60 ± 4.05)	−(5.20 ± 3.05)^a^	−(3.40 ± 1.25)*^,^**	24.19	<.01
LASr_c (%)	36.67 ± 11.74	24.70 ± 9.84^a^	5.10 ± 3.57*^,^**	28.73	<.01
LAScd_c (%)	−(15.33 ± 8.63)	−(10.65 ± 6.67)^a^	−(5.95 ± 3.66)*^,^**	15.17	<.01
LASct_c (%)	−(19.23 ± 10.33)	−(10.30 ± 6.68)^a^	−(4.25 ± 2.25)*^,^**	16.17	<.01

Compared with control group,

* *P* < .05; Compared with atrial fibrillation group without thrombus,

** *P* < .05.

LAEF = left atrial ejection fraction, LAEV = left atrial stroke volume, LAScd = left atrial conduit longitudinal strain, LAScd_c = left atrial conduit circumferential strain, LASct = left atrial contraction longitudinal strain, LASct_c = left atrial contraction circumferential strain, LASr = left atrial reservoir longitudinal strain, LASr_c = left atrial reservoir circumferential strain, LAVmax = left atrial maximum volume, LAVmin = left atrial minimum volume, LAVpreA = left atrial volume before atrial contraction.

### 3.4. Correlation analysis of left atrial LAEF and strain parameters in patients with AF

**Figure 3.**
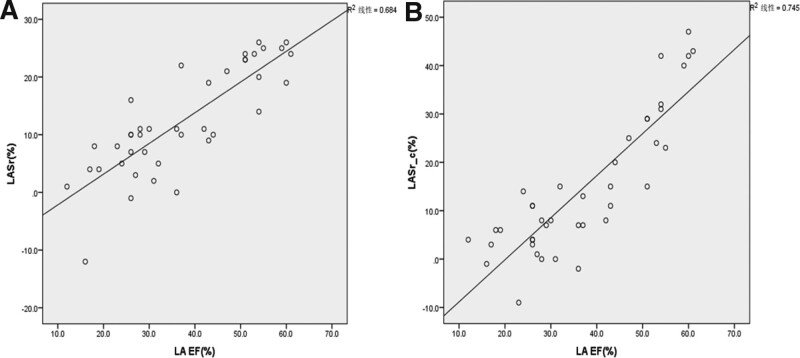
Correlation analysis of left atrial ejection fraction (LAEF) strain parameters in patients with atrial fibrillation (n = 40). (A) LAEF was associated with systolic left atrial longitudinal strain (LASr). (B) LAEF was associated with systolic left atrial circumferential strain (LASr-c).

Left atrial LAEF was positively correlated with LASr and LASr-c (*R*^2^ = 0.684, 0.745, both *P* < .01) and linearly correlated (Fig. 3).

## 4. Discussion

The function of left atrium in different stages of cardiac cycle is different: the left ventricle plays the function of blood storage during systolic period; At the early stage of diastole, the blood of pulmonary veins enters the left ventricle through the left atrium and plays a conduit function. In late diastole, blood from the atrium is pumped into the left ventricle to increase left ventricular filling, mainly for auxiliary pump function.^[4]^ Changes in the size and function of the left atrium are predictive factors and prognostic indicators of cardiovascular risk events, and are important factors in inducing AF.^[5]^The persistence of AF will lead to atrial remodeling. The increase of atrial myointerstitial fibrosis is mainly manifested as atrial enlargement in structure, and in function as reduced storage and catheter functions of the left atrium, reduced systolic function or even loss.^[6,7]^ Therefore, effective evaluation of left atrial capacity and function is of great clinical significance for patients with paroxysmal AF.

Real-time 3-dimensional spot tracking imaging (3D-STI) technology is designed to obtain the ability of deformation of each segment of myocardenum by calculating the strain and strain rate of each segment of myocardial movement.^[8]^ The EchoPAC 4D Auto LAQ software is based on volume parameters and provides strain parameters of the left atrium in different periods. It is a new quantitative analysis tool designed for the left atrium and can quickly and accurately evaluate the function of the left atrium.^[3]^ The myocardium of the left atrium is divided into deep myocardium and shallow myocardium. The shallow myocardium is transversally out of shape, while the deep myocardium is longitudinally and circumferentially out of shape, forming complex myocardium movements and generating radial, longitudinal and circumferential strains.^[9]^ Vieira MJ et al^[9]^ studied the mechanism of the left atrium through different imaging techniques and found that 3D-STI can be used to evaluate the function of the left atrium and is suitable for patients with AF. Badano et al^[10]^ concluded that 3D-STI technology was superior to 2-dimensional echocardiography in evaluating left atrial volume and function, especially in the case of left atrial enlargement, and the analytical results were in good agreement with MRI. Therefore, we selected 3D-STI technology to further analyze the data of left atrium in patients with AF.

Two-dimensional echocardiographic parameters among the 3 groups of subjects in this study showed that, with the progression of AF, LAd and LVd increased from the beginning and LVEF decreased slightly; after the occurrence of thrombosis in patients with AF, LAd and LVd increased significantly and LVEF decreased significantly. This is related to the remodeling of atrial cells in patients with a long history of AF in order to protect themselves, thus changing the structure of the atrium itself and further increasing the volume of the left atrium. Patients with AF have poor long-term ventricular rate control, and the renin-angiotensin system and sympathetic nervous system are activated, which leads to left ventricular remodeling.^[11]^ After left ventricular remodeling, the number of functional myocardial fibers in the left ventricle decreases, which in turn causes the decline of cardiac pumping function, and eventually leads to left ventricular dilatation and configuration changes. Therefore, as AF progresses over a long period of time, patients gradually develop left ventricular enlargement and reduced function.

In this study, the left atrial volume index and functional parameters of the 3 groups of subjects showed that the left atrial volume parameters of the group without thrombus increased compared with the control group, but not significantly, and the evaluated myocardial function parameters decreased. Compared with the group without thrombus, the atrial volume of the group with thrombus was further increased, and the evaluated myocardial function parameters were further reduced. It can be seen that in the early stage of AF, when the left atrial volume changes are not obvious, the left atrial storage function, the left atrial duct function and the active systolic function of the AF group without thrombosis have begun to decrease compared with the strain parameters of the control group. In the patients with AF, the atrium further expands and the atrial wall tension increases under long-term compensatory work, which eventually leads to the left atrial remodeling and the systolic function continues to decrease. Therefore, in patients with AF with left atrial appendage thrombosis, the atrial volume increased further, and the left atrial storage, duct and systolic functions continued to decrease significantly compared with those without AF. Previous studies on atrial function have suggested that the left atrium can inject 15% to 20% blood into the left ventricle, which is the key to ensuring left ventricular filling and normal stroke volume.^[12]^ In patients with AF, the atrium loses its auxiliary pump function, and blood accumulates in the left chamber, resulting in increased capacity, and long-term weakening of atrial muscle deformability and decreased active ejection function.^[13]^ Previous studies on atrial function believed that the left atrium can inject 15% to 20% blood into the left ventricle, which is the key to ensure the left ventricle filling and normal stroke volume. The left atrial ejection function continues to decrease, which in turn affects the left ventricular function. Therefore, in the course of the disease, patients begin to show clinical manifestations of right heart failure such as edema and dyspnea after the effective atrial contraction is disappeared.

LASr and LASr_c represent left atrial myocardial deformation during left ventricular systole, representing the diastolic capacity and compliance of the left atrium, and can reflect the storage function of the left atrium. The results of this study showed that the left atrial strain parameters LASr and LASr-c had a good correlation with LAEF in patients with AF, which could effectively evaluate the left atrial function.

Therefore, compared with 2-dimensional echocardiography, which can only measure the data in the standard section, 3-dimensional echocardiography can not only evaluate the left atrial volume and other related indexes, but also provide more obvious changes in myocardial function parameters sensitively during the progression of AF and give clues to the development of the disease.

LASr and LASr-c represent myocardial deformation of the left atrium during systolic period of the left ventricle, representing the diastolic capacity and compliance of the left atrium, and can reflect the storage function of the left atrium.^[14]^ The results of this study showed that the left atrial strain parameters LASr and LASr-c had a good correlation with LAEF in patients with AF, which could effectively evaluate the left atrial function.

Due to the small sample size of this study, the onset time and mode of AF in patients with AF were not analyzed in detail, so there are certain limitations. With the increase of sample size and the further improvement of 3D speckle tracking technology, the accuracy of the research results will be further improved.

In summary, it can be seen that the atrial muscle damage caused by paroxysmal AF in patients is a progressive process. With the progression of the disease, the volume of the left atrium in patients increases, and the storage, duct and contractile functions of the left atrium are gradually damaged. Three-dimensional spot tracking imaging (3D-STI) can effectively evaluate the changes of left atrial volume and function in patients with paroxysmal AF at different periods by obtaining left atrial volume and strain parameters, and can initially evaluate and predict the risk of complications in patients with paroxysmal AF, providing an important basis for clinical diagnosis and treatment.

## Author contributions

**Conceptualization:** Dong Bai.

**Data curation:** Dong Bai, Xiaojun Lu.

**Formal analysis:** Haijun Hou.

**Validation:** Lei Liang.

**Writing – original draft:** Yufeng Wang.
